# 

**Published:** 1918-09-21

**Authors:** 


September 21, 1918.] [Price Twopence

### The Hospital

The Workers' Newspaper of Administrative Medicine and Institutional Life, Administration, National Insurance and Health.

CONTENTS.

A State Medical Service in Detail ... ... ... 527

The Harvest-Time for Voluntary Hospitals ... 528

Hospital and Institutional News ... ... ... 529

The Coming Hospital Conference—Medical Men in Parliament—The Success of the Cardiff Appeal—A Hospital for the American Navy—War Emergency Formulæ—A Sequel to the Netley Protest—An Idle Threat and its Consequences—The Ancient Sherburn Hospital—A Chance for New Zealand Secretaries—A Test for Superspies—The Fall of the Curtain—The Sacrifice of the Civilian—Strike Fever in Asylums—Hospital Dispensers and Volunteer Service—An "Unbelievable" Occurrence—Advertising for Workhouse Inmates—A New Use for School Gardens—Hertfordshire Tuberculosis Report—Vindicating a Health Visitor—The Shortage of Probationers—This Week's Drug Market.

Papers on Madness ... ... ... ... ... 533

V. The Legal Aspect of Madness.

Case Records in a Teaching Hospital ... ... 534

New War Emergency Formulæ ... ... ... 535

Substitutes for Lard and Olive Oil.

The Correlation of Research ... ... ... ... 536

A Record of the Department of Health, New York.

The Metropolitan Asylums Board in 1917 ... ... 537

Its Work and Development.

CONTENTS.

The Nurse and Her Newspaper ... ... ... 538

The Matrons' and Sisters' Department ... ... 539

The Local Representative.

Round the Hospitals ... ... ... ... ... 539

Major Chapple on London Hospital Nurses—Sister-tutor Scholarships: The Lucky Three—The Coming of State Registration—Should Nurses Live Out?—Engagement on Written Recommendations—The Nursing Leaflet Competition.

The Editor's Letter-Box ... ... ... ... 543

London Hospital Nurses.
Matrons, and Nurse Agitators.
Should Nurses Live Out?

The Woman Worker's World ... ... ... 544

Mothers, Babies, and the Little Bill.

The Economic Value of the Penny ... ... 545

Hospital and Institutional Results ... ... ... 545

The Order on Parasitic Mange ... ... ... 545

The Public Health ... ... ... ... ... 546

Australian Notes ... ... ... ... ... ... 546

Matrons' and Sisters' Appointments ... ... ... iv

Institutional Worker ... ... ... ... ... I

A Burning Question.

